# Hyaluronate Nanoparticles as a Delivery System to Carry Neuroglobin to the Brain after Stroke

**DOI:** 10.3390/pharmaceutics12010040

**Published:** 2020-01-03

**Authors:** Santos Blanco, Sebastián Peralta, María Encarnación Morales, Esther Martínez-Lara, José Rafael Pedrajas, Herminia Castán, María Ángeles Peinado, María Adolfina Ruiz

**Affiliations:** 1 Department of Experimental Biology, University of Jaén, Building B3, Campus de Las Lagunillas s/n, 23071 Jaén, Spain; sblanco@ujaen.es (S.B.); elara@ujaen.es (E.M.-L.); pedrajas@ujaen.es (J.R.P.); 2 Department of Pharmacy and Pharmaceutical Technology, School of Pharmacy, University of Granada, Campus de Cartuja s/n, 18071 Granada, Spain; seperaltag@gmail.com (S.P.); maen@ugr.es (M.E.M.); castan@correo.ugr.es (H.C.)

**Keywords:** neuroglobin, nanoparticles, stroke, sodium hyaluronate

## Abstract

Therapies against stroke can restore the blood supply but cannot prevent the ischemic damage nor stimulate the recovery of the infarcted zone. The neuroglobin protein plays an important role in the neuro-regeneration process after stroke; however, the method for its effective systemic application has not been identified yet, as neuroglobin is unable to pass through the blood-brain barrier. Previously, we developed different types of sodium hyaluronate nanoparticles, which successfully cross the blood-brain barrier after stroke. In this work, these nanoparticles have been used to carry neuroglobin through the bloodstream to the nerve cells in rats submitted to stroke. We have biosynthesized rat-recombinant neuroglobin and determined the formulation of sodium hyaluronate nanoparticles loaded with neuroglobin, as well as its size and ζ-potential, encapsulation efficiently, in vitro release, and its kinetic of liberation. The results show that the formulation achieved is highly compatible with pharmaceutical use and may act as a delivery system to transport neuroglobin within the blood. We have found that this formulation injected intravenously immediately after stroke reached the damaged cerebral parenchyma at early stages (2 h). Neuroglobin colocalizes with its nanocarriers inside the nerve cells and remains after 24 h of reperfusion. In conclusion, the systemic administration of neuroglobin linked to nanoparticles is a potential neuroprotective drug-delivery strategy after stroke episodes.

## 1. Introduction

Stroke is a leading cause of death and long-term disability worldwide [1,2]. Ischemic stroke occurring in the zone irrigated by the middle cerebral artery, it can affect the parietal cortex and striatum, which suffer an energetic unbalance leading to neuronal and glial damage and even to cell death [3]. The damaged zone, or infarct, has a core and a penumbra, this latter being capable of recuperation [4]. However, current treatments, thrombolysis and thrombectomy are not effective enough due to the narrow therapeutic window and complexity of administration [5,6,7]. None of these treatments provide neuroprotection to the ischemic tissue damaged during the reperfusion period. Numerous clinical trials for neuroprotectants against stroke and neurodegenerative disorders have been conducted, reporting unsatisfactory outcomes [8,9]. Accumulating evidence has demonstrated that the oxygen-binding neuroglobin protein (NGB) [10], acts as a neuroprotective molecule against stroke in humans [1,2,11], and against some hypoxic/ischemic (H/I), and oxidative stress-related insults in animals and cultured neurons [12,13,14,15,16,17]. Overexpression of NGB in rat brains reduced the infarct size following transient middle cerebral artery occlusion (tMCAO) [18], and in an ischemic pre-conditioning model of MCAO [19]. The inhibition of the expression of NGB with NGB-antisense oligodeoxynucleotide eliminates the neuroprotective effect mediated by hypoxic post-conditioning, whereas overexpression of NGB ameliorates neuronal damage in CA1 after transient global ischemia, indicating that hypoxic post-conditioning confers neuroprotective effects via upregulation of NGB [20]. NGB has also been identified as a factor driving for axon regeneration after ischemia/reperfusion, correlating positively with the elevation of axon-regeneration markers like growth-associated protein 43 (GAP43), neurofilament-200, and Tau-1. Upregulation of NGB does not appear until 48–72 h of reperfusion after the ischemic insult [21,22]. These data, together with NGB being expressed at physiological levels, do not enable resistance to neuronal death following ischemia [23,24]. As such, effective and innocuous therapies allowing an increase of NGB in the damaged cerebral parenchyma immediately after the ischemic insult must be developed. Therefore, rapidly increasing NGB in the penumbra zone of the infarct could change the prognosis of stroke.

Although the goal of increasing NGB in the cerebral parenchyma after ischemia may appear feasible, i.e., injecting NGB at a systemic level, both the immune response and the selectivity of the blood-brain barrier (BBB) prevent the entry of large molecules to the brain and represent considerable obstacles that need to be overcome. Several studies using delivery systems such as viral vectors [18] or cell-penetrating peptides (CPP) [1], tried to solve problems, but both approaches presented a series of inconveniences [21], which make their use unfeasible in humans. Given these difficulties, we developed different types of sodium hyaluronate (SH) nanoparticles (NPs) to protect and transport large molecules, like NG,) from blood to the brain after a stroke episode [25]. Particularly, we used different gelation methods and envelopes to synthesize innocuous and functionalized NPs, which may act as Trojan horses able to protect and help their cargo to cross the BBB, thus leading NGB to the damaged nerve cells on time. We characterized these NPs physicochemically and conducted in vitro and in vivo studies to find the NPs with the best ability to reach the target cells. Factors such as lipophilicity or size of the NPs are important to determine its permeability, but other parameters such as the ζ-potential are also decisive, due to the highly negative electrokinetic membrane potential of the endothelial cells. These NPs are synthesized from the natural polymer sodium hyaluronate, which has the ability to gelify in the presence of a cross-linker without the presence of organic solvents or high temperatures. This feature conveniently avoids the damage or loss of labile drugs [26] like NGB. Moreover, these NPs have the advantage of being biocompatible and promoting biological signals that interact with specific receptors of endothelial cells [27]. Facilitating the infiltration of NPs into the central nervous system, even in the absence of lipophilicity. Consequently the drug transported by the NPs can easily reach its final target: The hypoxic damaged nerve cells.

Naked NPs in the bloodstream are quickly removed due to opsonization and capture by the reticuloendothelial system, even though they have suitable composition and size. Therefore, any strategy that would increase the lifespan of NPs in the blood should be an objective in the design of NPs [28]; the adsorption of surfactants on the NPs surface has been shown to enhance its life expectancy in the circulation [29]. Consequently, we used polysorbate 80 [30] to achieve this goal, as previous studies conducted in vivo demonstrated that the adsorption of polysorbates, like polysorbate 80, on the surface of the NPs leads to changes in the distribution of the NPs [31]. The penetration of NPs into cells occurs through the stimulation of receptor-mediated endocytosis, particularly through the interaction of NPs with LDL receptors on the surface of brain endothelial cells. It was concluded that the reason for such interaction was the adsorption of endogenous LDL from plasma to polysorbate 80 [32].

Chitosan (CH), a natural biocompatible cationic polysaccharide used as an excipient in several applications related to drug administration [33,34,35,36,37], has been postulated to be a good candidate for NPs coating due to its ability to increase the positive charge density of NPs, which favors the permeation of the NPs through the endothelial cells of the BBB, to lengthen the half-life of NPs in plasma, or to facilitate the bio-adhesion to the endothelium after electrostatic interaction [38].

Given this background, in this study, we developed a series of procedures, which range from the biosynthesis of recombinant NGB to the preparation of functional NPs, to produce an innocuous neuroprotective formulation able to bring NGB from blood to the damaged nervous cells quickly. We used an animal model of transient middle cerebral artery occlusion (tMCAO) and confocal microscopy to verify that these NPs loaded with NGB reach their therapeutic targets and that NGB is released in the infarcted area of the cerebral parenchyma. The brains of animals submitted to tMCAO were checked to detect the NPs loaded with NGB (NGB–NPs) using confocal microscopy at 2 h and 24 h after the onset of reperfusion. Finally, to improve the efficiency of the NGB–NPs, we completed lyophilization tests and stability studies of NGB to preserve the formulation and verify its therapeutic ability over time.

## 2. Materials and Methods

### 2.1. Materials

Sodium hyaluronate (SH) (1.0–1.5 MDa) was purchased from Fagron (Barcelona, Spain), and TALON™ metal affinity column from Takara Bio Europe (Saint-Germain-en-Laye, France). Grape seed oil and Tween^®^ 80, as well as Freund’s adjuvant, were obtained from Guinama (Valencia, Spain) and Fisher Scientific (Madrid, Spain), respectively. Calcium chloride, chitosan (low molecular weight), glacial acetic acid, glycerol tripalmitin, rhodamine-123, lysozyme, formalin, DNase I, normal goat serum, DAPI, Triton X-100 and LB medium were obtained from Sigma Aldrich (Madrid, Spain). Dichloromethane and ampicillin were purchased from VWR (Barcelona, Spain), ketamine (Imalgene 100 mg/mL) from Merial Laboratorios S.A. (Sant Cugat del Vallès, Spain), and xylacine (Rompun^®^) from Bayer (Sant Joan Despí, Spain). Uranyl acetate was obtained from Electron Microscopy Sciences (Hatfield, PA, USA), and distilled water was prepared using a Millipore (Billerica, MA, USA) system. Sevoflurane was purchased from Abbot Laboratories (Madrid, Spain), O.C.T medium from Sakura (Alphen aan den Rijn, Netherlands), Cy2-linked goat anti-rabbit IgG from Pierce (Rockford, IL, USA), and IPTG from Canvax Biotech (Córdoba, Spain).

### 2.2. Expression and Purification of Recombinant Neuroglobin

The pET15b/NGB expression vector was constructed by Genscript Biotech Corp. The NCBI Reference Sequence NM_033359.3 that codes for rat neuroglobin was inserted between the Nde I and BamH I sites of the pET15b plasmid. *E. coli* BL21(DE3) transformed with pET15b/NGB construct were inoculated in LB medium containing 0.1 mg ampicillin/mL and grown at 37 °C until A600 = 0.8. Then, the expression of the recombinant protein was induced by the addition of 0.5 mM isopropyl-1-thio-β-D-galatopyranoside (IPTG), and growth was continued at 37 °C for 4 h. Cells were collected by centrifugation and suspended in 20 mM Tris-HCl, pH 8; 0.1 M NaCl (Tris buffer) containing 0.5 mg lysozyme/mL and 1 μg DNase I/mL. Then, cells were lysed by sonication and the homogenate was clarified by centrifugation at 10,000× *g* for 30 min. The cell-free extract was filtered and loaded onto a TALON™ (Takara Bio Inc., Kusatsu, Japan) metal affinity column equilibrated with Tris buffer. The column was washed with this same buffer containing 5 mM imidazole and the recombinant His-tagged NGB protein was eluted with 100 mM imidazole. To separate the His-tag from the NGB protein, 1 unit of thrombin/mL was added to the purified recombinant protein, incubated for 2 h at room temperature, and dialyzed against Tris buffer. Finally, the sample was passed through the TALON™ metal affinity column equilibrated with Tris buffer, and the flow-through containing NGB protein was collected. Protein concentration was determined according to the molar extinction coefficient at 280 nm for NGB (22,500 M^−1^·cm^−1^).

### 2.3. Preparation of Antiserum against NGB

The polyclonal NGB antiserum was produced in the experimentation animal service of the University of Cordoba (SAEX, Cordoba, Spain) using New Zealand rabbits. All procedures were previously approved (approval # 23/05/2016/090, 23 May 2016) by the local Animal Care Committee and performed in compliance with the Spanish legislation and in accordance with the European Union (EU) Directive 2010/63/EU (2010).

For antibody production, rabbits were injected weekly subcutaneously at multiple sites with recombinant NGB. 250 μg of protein were mixed with complete Freund’s adjuvant for the primary immunization and 125 μg in incomplete Freund’s adjuvant for the subsequent immunizations. Blood was extracted from the immunized animals and centrifuged at 20,000× *g* for 30 min. The supernatant was heated at 56 °C to inactivate the complement proteins and the resulting serum was used as a polyclonal probe for immunoassays. Serum extracted prior to immunization was also obtained as a negative control.

### 2.4. Synthesis of NPs with NGB

The emulsification and external gelation methodology used to produce NPs is based on the formation of a water-oil emulsion (W/O), as previously reported with slight modifications [25]. To obtain this type of NP, an initial aqueous phase was elaborated as follows: 0.26% (*w*/*v*) SH and 2.6% (*w*/*v*) Tween^®^ 80 (Bio-Rad Laboratories, S.A., Alcobendas, Madrid, Spain) were dispersed in 25 mL of 10^−4^ M rhodamine solution under magnetic stirring for 10 min at 200 rpm. Then, 35 mg of the purified recombinant NGB diluted in PBS was added to the previous solution, stirring for further 10 min. Afterward, 90 mL of grapeseed oil was added at a speed of 1200 rpm for 10 min. Next, 3 mL of distilled water with 0.25 g of calcium chloride was incorporated to the dispersion at a speed of 1600 rpm for about 10 min, continuing the stirring at 1200 rpm for 2 h. Afterward, the oil phase was eliminated by centrifugation at 10,000 rpm (5× *g*) for 15 min. Once this dispersion of NPs with NGB was optimized, they were coated with CH and glycerol tripalmitin (GT); GT is a biocompatible triglyceride present in the human body as fat [39]. The procedure was made as follows: 1 mL of 1% glacial acetic acid was added to 1% CH solution (*v*/*v*), as this acidification facilitated the dissolution of CH before coating. Then, this CH solution was mixed in equal parts with the dispersion of the NPs, keeping the mixture in magnetic stirring for 2 h; once coated, the remaining CH was easily removed after centrifugation at 10,000 rpm (5× *g*) for 35 min. Later, the resulting CH-coated sodium hyaluronate NPs containing NGB were subjected to a new coating with GT, because its lipid nature facilitated the penetration of the NPs through the BBB. For that, 0.1% GT was dissolved in dichloromethane (*w*/*v*), added to 10 mL of the colloidal dispersion of NGB–NPs, and maintained under magnetic stirring until complete evaporation of the solvent.

### 2.5. Characterization of NPs

#### 2.5.1. Particle Size and Polydispersity Index (PDI)

The size and PDI of the NGB-NPs were determined using a laser scattering particle size analyzer at 25.0 °C ± 0.5 °C (Malvern Zetasizer Nano ZS^®^; Malvern Instruments Ltd., Malvern, UK). Dispersions of NGB–NPs in deionized water was prepared and the average size was expressed in nanometers (nm).

#### 2.5.2. Zeta Potential (ζ-Potential) Measurement

ζ-potentials of NGB–NPs were also determined using electrophoretic light scattering and a molecular weight analyzer with static light scattering (Malvern Zetasizer Nano ZS^®^) by dispersing NGB–NPs in deionized distilled water. Samples were placed in a capillary cell, and ζ-potentials of these NGB–NPs were measured and are expressed in millivolts (mV).

#### 2.5.3. Transmission and Scanning Electron Microscopy (TEM and SEM) Analysis

For TEM images of NGB–NPs, the samples were prepared with negative staining. The dispersion was incubated in a grid with a carbon support film for 5 min in a Petri dish and contrasted with 1% uranyl acetate in aqueous solution. Then, these samples were mounted and visualized on a TEM microscope LEO 906E (Carl Zeiss LEO 906E, Jena, Germany).

For SEM images, a drop of the colloidal dispersion of NGB–NPs was deposited on the corresponding support for the FESEM (pin stub mount, Hitachi Ltd., Chiyoda, Tokyo, Japan), allowing it to dry at room temperature. Subsequently, it was sputtered with carbon by means of a carbon coater (Polaron CC7650, Quorum Technologies Ltd., Laughton, East Sussex, UK) and visualized on a SEM microscope (Hitachi S-510, Hitachi Ltd., Chiyoda, Tokyo, Japan).

Both microscopes are located at the Scientific Instrumentation Center of the University of Granada (Granada Spain).

#### 2.5.4. High Performance Liquid Chromatography (HPLC) Conditions

An Acquity UPLC System instrument (Waters Technologies Corporation, Milford, MA, USA) equipped with autosampler, column temperature controller, TUV detector, quaternary pump, and an online degasser, was used for the analyses. A Waters UPLC™ (Waters Technologies Corporation, Milford, MA, USA), thermostatted at 25 °C, was used for chromatographic separations, a Waters UPPLC™ Protein BEH C4 2.1 × 50 mm, 1.7 µm column was used. The mobile phase was formed by 2 channels: A (water) and B (acetonitrile). The gradient elution program was: 5% B, T_5_: 95% B, T_6_: 100% B, T_6.1_: 5% B. The flow rate was 0.2 mL/min with detection wavelength of 290 nm. The injection volume was 10 μL and each run was followed by an equilibration period of 1.9 min. The total run time was 8 min and the whole method was validated.

### 2.6. Determination of Drug Encapsulation Efficiency (DEE, %)

To determine the efficiency of the encapsulation of NGB–NPs, the dispersion was centrifuged at 10,000 rpm (Eppendorf AG 5804; Hamburg, Germany) for 20 min until a solid precipitate was obtained. The NGB content in the supernatant was analyzed by HPLC under the conditions described above, and the encapsulation was determined using the following formula: encapsulated percentage = (actual drug content/theoretical drug content) × 100.

#### 2.6.1. In Vitro Drug Release Study

An in vitro NGB release study was performed using a dialysis membrane (50 kDa molecular weight cut off; Spectra/Por^®^ 6, Spectrum Chemical Mfg. Corp., New Brunswick, NJ, USA) containing 25 mL of phosphate buffer at pH 7.4. Dispersion of NPs (1.6 mL) were placed in the dialysis membrane and both ends were sealed with magnetic weighted closures (Spectra/Por Clousures^®^, Sigma, Madrid, Spain). Then, the dialysis bag was maintained in the receptor compartment containing dissolution medium (phosphate buffer pH 7.4) at 37 ± 0.5 °C and stirring at 100 rpm for 72 h. At regular time intervals, 0.8 mL of the sample was withdrawn and replaced with freshly prepared phosphate buffer pH 7.4. The NGB content in the samples was analyzed by HPLC under the conditions described above.

#### 2.6.2. In Vitro Drug Release Kinetics

Different mathematical models were tested to choose the one that best explained the release kinetics. Data obtained from the in vitro analysis of the drug release were submitted to the Akaike Information Criterion (AIC), one of the most widely used methods for the determination of the model that best explains the diffusion process [40]. AIC allows to find the function that more accurately fits to the drug release process. The criterion identifies the model that best fits the data as the one with the minimum value of AIC, and was calculated by applying the following equation:

AIC = n × ln SSQ + 2p

where n is the number of pairs of experimental values, ln is the Neperian logarithm, SSQ is the Sum of residual squares, and p is the number of parameters of the adjustment function.

### 2.7. Stability Studies

#### 2.7.1. Lyophilization of NGB

Lyophilization was conducted using a freeze dryer (Telstar^®^ Cryodos 50, Telstar Spain, Tarrasa, Barcelona, Spain). First, 1 mL sample vials of 2.04 mg/mL of recombinant NGB in PBS were frozen at −80 °C for 24 h. The vials were subjected to vacuum (less than 500 mbar) until the end of the process and obtained a residual humidity of less than 5%. The lyophilized samples were kept at 6 °C until reconstitution and measured by HPLC.

#### 2.7.2. Lyophilization of NGB–NPs

The dispersion of NGB–NPs was lyophilized using a freeze dryer (Telstar^®^ Cryodos 50), prior to freezing at −80 °C for 24 h. Then, vials were subjected to vacuum (less than 500 mbar) at the end of the process, obtaining a residual humidity of less than 5%. Afterward, the integrity of the NPs linked to NGB was easily redispersed and analyzed by means of a TEM microscopy and a Zetasizer^®^ (Malvern Panalytical B.V., San Sebastián de los Reyes, Madrid, Spain)

#### 2.7.3. Stability to 6 °C

A solution of NGB in water was stored at 6 °C for 3 months and measured by HPLC.

### 2.8. In Vivo Studies

Adult male Wistar rats (Charles River) were used to analyze the penetration of NGB–NPs into the cerebral parenchyma. Experimental procedures were supervised by the Animal Care Committee of the University of Jaén and performed in compliance with the Spanish legislation and in accordance with the EU Directive 2010/63/EU (2010). Animal studies were performed in the Animal Production and Experimental Centre of the University of Jaén (CPEA) (approval # 23/05/2016/090, 23 May 2016).

Animals were subjected to a stroke model of tMCAO and euthanized at 2 h (*n* = 8) or 24 h (*n* = 8) after the onset of reperfusion to monitor the location of NGB–NPs at these earlier stages of the ischemia/reperfusion injury, while the main molecular events involved in ischemic damage were occurring [41]. All animals were injected intravenous (1 mL/kg body weight) with the formulation of SH-NPs linked to NGB (2.15 mg/mL) immediately after tMCAO throughout the lateral vein of the tail.

#### 2.8.1. Stroke Model

**Figure 1:**
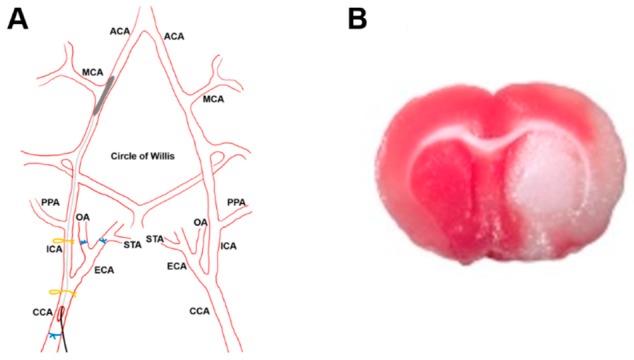
Stroke model. (**A**): Schematic representation of the stroke model of transient middle cerebral artery occlusion (tMCAO). CCA (Common Cerebral Artery); ECA (External Cerebral Artery); ICA (Internal Cerebral Artery); MCA (Middle Cerebral Artery). (**B**): Representative image of the brain of a rat summited to tMCAO stained with TTC, showing the infarcted zone (white) within the right hemisphere.

The stroke model was applied using a slight modification of the model previously described [42,43]. Briefly, a 23 mm segment of a 3-0 nylon monofilament suture with a 3–4 mm coating (Doccol Corporation, Redlands, CA, USA), was inserted through the right common carotid artery and advanced until occluding the origin of the middle cerebral artery. The suture was removed after 45 min of occlusion [25]. Only animals with a cerebral blood flow reduction over 60%, evaluated by a Laser-Doppler flow probe (tip diameter 1 mm) attached to a flowmeter (moorVMS-LDF1, Moor Instruments, Axminster, UK) were included in the study (Figure 1A).

#### 2.8.2. Histology and Microscopy

After euthanasia, animals were perfused through the left ventricle with 0.01 M phosphate-buffered saline (PBS, pH 7.4) followed by 300 mL of 10% formalin. The brains were removed and post-fixed for a further 4 h in the same fixative at room temperature. Samples were cryoprotected by immersion overnight at 4 °C in 0.1 M PBS containing 30% sucrose and then embedded in O.C.T. medium to obtain serial rostrocaudal sections (20 µm thick) using a cryostat (Leica CM1950; Leica Microsistemas S.L.U., L’Hospitalet de Llobregat, Spain). Free-floating sections were then washed 3 times in PBS for 5 min each, and incubated in blocking solution containing 0.1% Triton X-100 and 3% normal goat serum (NGS) in PBS for 2 h at room temperature; afterward, sections were incubated in rabbit anti-NGB antisera 1:500 in PBS containing 0.2% Triton X-100, overnight at 4 °C. After several rinses in PBS, the sections were incubated with Cy2-linked goat anti-rabbit IgG 1:1000 (PA42004, GE Healthcare, Little Chalfont, UK). After 3 rinses in PBS, the sections were stained with 4′,6-Diamidino-2-phenylindole, dihydrochloride (DAPI) (124653, Merck, Darmstadt, Germany) at 1:2000 for 8 min to visualize the cell nuclei. Then, the sections were quickly dehydrated in a graded ethanol series, cleared, and mounted on slides for examination under confocal laser scanning microscope (Leica TCS SP5 II; Leica Microsistemas S.L.U., L’Hospitalet de Llobregat, Spain) in the Central Research Support Services of the University of Jaén (SCAI). Cell nuclei were visualized in blue due to DAPI staining (excitation 358 nm, emission 529 nm), SH–NPs appeared in red due to rhodamine (excitation 507 nm, emission 525 nm), and NGB in green due to the Cy2 immunofluorescence staining (excitation 492 nm, emission 510 nm). To ensure that the signal detected in each channel was not due to autofluorescence, TrueVIEW Autofluorescence Quenching Kit (Vector Laboratories, Burlingame, CA, USA) and control samples of rats not injected with NPs–NGB were used. Thus, the biodistribution of SH–NPs and NGB within the infarcted area of the brains could be histologically visualized. Some sections of the infarcted brains were stained with triphenyltetrazolium chloride monohydrate (TTC) [44] in order to visualize the infarcted area, which appeared white in contrast to the red-stained normal areas (Figure 1B). 

#### 2.8.3. Fluorescence Quantification in the Cerebral Parenchyma

**Figure 2:**
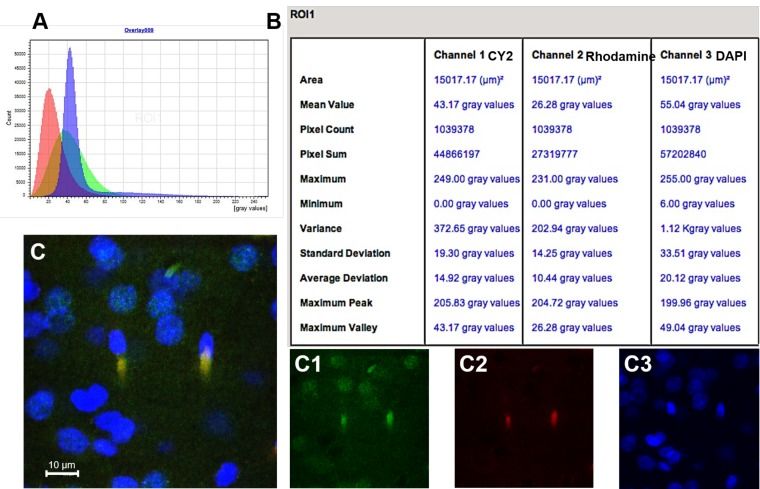
The methodology used to quantify the fluorescence emitted by the different fluorophores used to label NGB, NPs, and cell nuclei in histological sections of the infarcted cerebral parenchyma. A and B: Graphic representation (**A**) and semiquantitative data (**B**) of the mean grey values obtained by the confocal software AF (Leica) from fluorophores Cy2 (channel 1), rhodamine (channel 2), and DAPI (channel 3). (**C**): Merged image from a histological section of the infarcted parietal cortex, as the result of the overly of images (**C1**–**C3**), which, respectively, show Cy2, rhodamine, and DAPI fluorescence separately: Cell nuclei appear in blue (DAPI); neuroglobin (NGB) in green (Cy2) and nanoparticles (NPs) in red (rhodamine) are colocalized (light orange/yellow) in the cytoplasm of the nervous cells.

The fluorescence of the rhodamine, Cy2 and DAPI were automatically evaluated in different brain sections using the Leica LCS software (Leica Microsystems, Wetzlar, Germany). Using this software, that analyses the fluorescence emitted by each fluorophore (mean grey value) from areas previously determined in the histological sections, a semiquantitative estimation of the presence of NPs (rhodamine fluorescence), NGB (Cy2 fluorescence), and cell nuclei (DAPI fluorescence) located in the cerebral parenchyma of the brains submitted to tMCAO at reperfusion periods of 2 h and 24 h. Data from different histological sections in areas located at the same level of the ischemic parietal cortex in both groups of animals were obtained strictly under the same optical conditions and later recorded for further analysis (Figure 2). The data 2 h and 24 h were compared using the Student’s *t*-test with a 95% confidence level.

## 3. Results and Discussion

### 3.1. Physicochemical Characterization of NGB–NPs

#### 3.1.1. Particle Size and PDI

**Figure 3:**
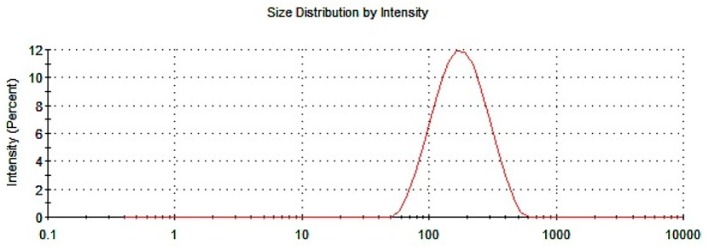
Size distribution of NGB–NPs.

The size of the particles is a parameter highly related to the functionality of the NPs, which is directly connected to the appearance of the dispersion of NPs. In this sense, the macroscopic analysis shows the absence of aggregates or sediments. It presents turbidity and a white hue. According to the information previously provided by other authors [45], NPs with a size between 100 and 200 nm were easily captured by endocytosis, and particles greater than 300 nm were captured by phagocytosis [45]; therefore, the ideal size of the NPs linked to NGB should be below 300 nm. Table 1 shows the results obtained for the different measurements made on the dispersion of NGB–NPs: As seen, the average size was below 150 nm. According to the data analyzed, this size must be a consequence of NGB, which exerts a clear influence on this parameter with respect to the average size of the NPs without the drug, as we have previously published [25]. Even so, this size, alongside other parameters exhibited by the NGB–NPs (Table 1) was exceedingly acceptable for its functionality. On the other hand, polydispersion, a value inherent to the elaboration of the NPs, presented low values, which was indicative of the quality and homogeneity of the preparation. Figure 3 represents the size distribution of NGB–NPs by intensity. As can be seen, the data showed a unimodal size distribution, the fact that confirmed the previous. There was a predominance of medium-sized NPs and no other peaks of intensity were observed, confirming the absence of large aggregates.

#### 3.1.2. Zeta Potential

ζ-potential measure provides important information about the aggregation and stability of NPs [46,47]. In our case, compliance with Fick’s law is essential to allow NPs to cross through cell membranes [48]. NGB–NPs have positive ζ-potential values close to 30 mV (Table 1). The positive charge is a decisive factor in the penetration of the NPs through the BBB, due to the presence of proteoglycans, mucopolysaccharides, glycolipids and glycoproteins with sulfates and sialic acids; these compounds confer a high negative charge at physiological pH to the cell plasma membrane. Thus, the electrostatic interactions between the positive charge of the NPs and the negative charge of the cell membrane could favor its adsorption and transport throughout the endothelial cells [49]. Some NPs with moderate (up to 15 mV) or high values of positive ζ-potential (above 15 mV) can cross the BBB, providing efficient systems of administration of drugs to the brain [50,51].

According to the sizes obtained and their ζ-potential, the sample results were stable and did not form agglomerates as evidenced by the analyses. As discussed above, the analysis of polydispersity (Table 1) showed a curve with a unimodal size distribution (Figure 3). Therefore, the ζ-potential influences on the stability of the dispersion.

#### 3.1.3. TEM and SEM Analyses

**Figure 4:**
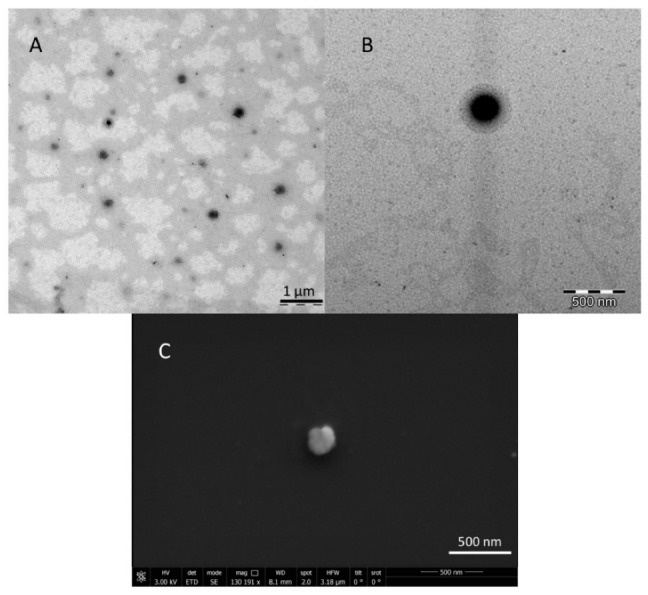
Microscopy characterization of NGB–NPs. (**A**) Field with different NGB–NPs at a scale of 1 µm (TEM). (**B**) Amplified field with a unique NP–NGB (500 nm) (TEM). (**C**) Microphotograph showing a unique NGB–NP with a 500 nm scale (SEM).

Representative microscopic images of NGB–NPs are presented in Figure 4A,B (TEM) and Figure 4C (SEM). Figure 4A shows a general perspective of the NP dispersion using TEM, where different isolated NPs with homogeneous size can be observed. These observations can be related to the values obtained in the physicochemical analysis. Each NP was visualized under TEM with a central core and a slightly irregular surrounding halo that could be assigned to the coatings of the NPs and to the encapsulation of the protein. In Figure 4B, an isolated NP shows a fairly defined and spherical shape. As previously reported, this spherical shape could serve for the maintenance of the textural quality [52,53].

The SEM image in Figure 4C shows another isolated NP. Although its silhouette was spherical, it presented some typical irregularities due to the coatings and encapsulation of NGB. Its surface was not smooth but rough and spongy. A positive sign of functionality was that pores are not evident on the NP surface. High porosity was associated with a fast and easy diffusion of water and other fluids in and out from the matrix, subsequently affecting the integrity and stability of the NPs, which would not guarantee the protection and transport of the NGB [54,55].

### 3.2. Drug Encapsulation Efficiency (DEE, %)

DEE is a fundamental parameter for NPs, as well as for the drug loading (NGB). Due to the encapsulation, efficiency must be high enough to minimize the amount of formulation and maximize preparation performance. The dose must also match the action of the drug. To our knowledge, no reports exist of using NPs to deliver NGB to the ischemic brain, although strategies using viral vectors [18] and cell penetrating peptide (CPP) [1] had already been tested for this purpose. In mice suffering from a brain injury as a consequence of the subarachnoid hemorrhage, the dose of injected CPP–NGB was 6 mg/kg [56]. This dose and even higher doses applied in some stroke models [1] seemed to be too high, as described in reference [21]. Therefore, we used a dose based on maximum encapsulation, which in our case was close to the values for its use in vivo. The data obtained from HPLC analysis were promising, with DEE values of 92.37% ± 0.84%. This favorable result can be attributed to the external gelling inside an emulsion since both the polymer and the protein have a tendency for the aqueous phase.

#### In Vitro NGB–NPs Release

**Figure 5:**
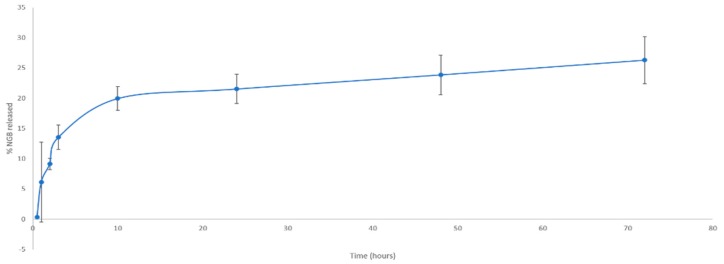
In vitro release curve (%) of the encapsulated NGB transferred by the NGB–NPs in the function of time (over 72 h) in the study.

The drug release pattern is a crucial parameter drug delivery carrier, including polymeric NPs. In most cases, the polymers and materials used for the coating determine the release rate of the drugs, as previously reported [57]. Figure 5 shows the percentage of protein released from 0 to 72 h. During the first 10 h, most of the protein was discharged into the medium, mainly due to the release of the protein on the surface and near the outer edge of the NPs. Then, the release was maintained in a more sustained manner over time, reaching its maximum at 72 h; this second wave of liberation from 10 to 72 h corresponds to the release of the protein encapsulated inside the NPs, which could justify the flatter pattern found in this period. No burst effect was observed, maybe due to the presence of the coatings, especially to the lipids which act as a sort of membrane.

The experimental data were adjusted to each of the mathematical methods using the Akaike discriminatory criterion (AIC), where the function with the lowest AIC value better explains the diffusion process. Table 2 shows the data obtained after the application of the method. The cubic root kinetics, which was related to the diffusion speed studies, was due to geometrical reasons, the best value, specifically, when the NPs with the drug had a spherical shape or in the presence of surfactants that formed micelles in the solution. In this case, the variation that presents the surface of the solid was a function directly related to the cubic root of the square of the volume of the NPs [40], as expressed in the following equation:

$$S=K\cdot \sqrt[{3}]{V^{2}}$$

### 3.3. Studies of Stability of NGB and NPs

To assess the stability of the NGB and the NGB–NPs for their use as a pharmaceutical formulation, both were tested separately in stability studies at 6 °C, and lyophilized.

Concerning NGB, once it was lyophilized, the samples were analyzed by HPLC. Table 3 shows the average of the percentage of NGB concentrations of different aliquots with respect to a non-lyophilized standard from which all the samples were taken. The percentage results, which did not reflect any significant loss during the process, indicated that NGB was practically entirely preserved. In fact, the NGB powder recomposed at several times of 0, 15, and 30 days showed very good stability, as NGB losses were negligible and the protein had almost no degradation.

The stability of the NGB preserved at 6 °C was also measured over time by HPLC. Table 4 displays the average of 3 aliquots measured over 3 months. The results showed a degradation of NGB over this period, with a total loss of almost 25% of the initial concentration.

**Figure 6:**
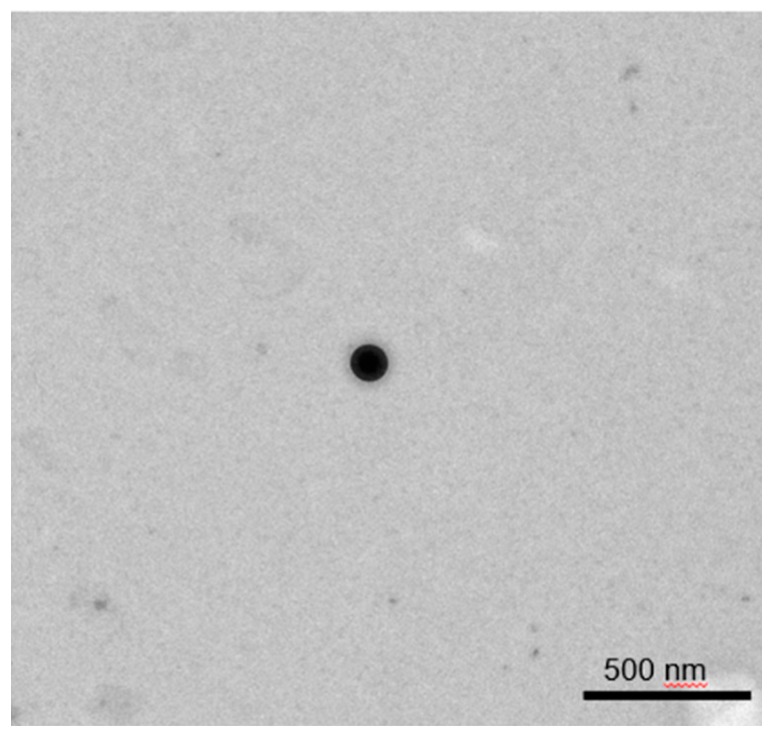
TEM microphotography of a representative lyophilized NP.

Once the lyophilization of NGB was evaluated, we proceeded to lyophilize the NPs alone to study its stability. To achieve this goal, the size and morphology of NPs were assessed by microscopy. No significant changes in size were appreciated after the lyophilization process, as very similar values were found with or without lyophilization (Table 5). Regarding TEM analysis, Figure 6 shows a representative image of a NP showing how the NPs maintained its constitution after lyophilization with a spherical and defined shape, showing once more no substantial changes as a consequence of this procedure.

### 3.4. In Vivo Studies: Penetration of NGB–NPs into the Cerebral Parenchyma

Previous extensive tests of nanoparticle cytotoxicity were performed in cell cultures (results not showed); these tests showed that nanoparticles were biocompatible, biodegradable, non-toxic, and non-immunogenic. On the other hand, NGB is an endogenous protein and numerous studies prove its zero toxicity. In this way, NGB exerts a relevant action in the protection against the toxicity of nitric oxide [58], H_2_O_2_ [59], paraquat [59] and β-amyloid [60].

**Figure 7:**
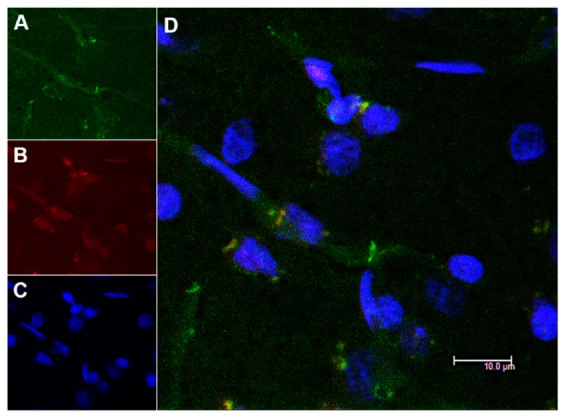
Confocal microscopy images of the parietal cortex of animals submitted to tMCAO after 2 h of reperfusion. (**A**): Cy2 green fluorescence represents NGB. (**B**): Rhodamine red fluorescence detects NPs. (**C**): DAPI blue fluorescence marks cell nuclei. Images are representative microphotographs of samples from 8 experimental animals. (**D**): Merge of images of (**A**–**C**). Scale bar: 10 μm.

**Figure 8:**
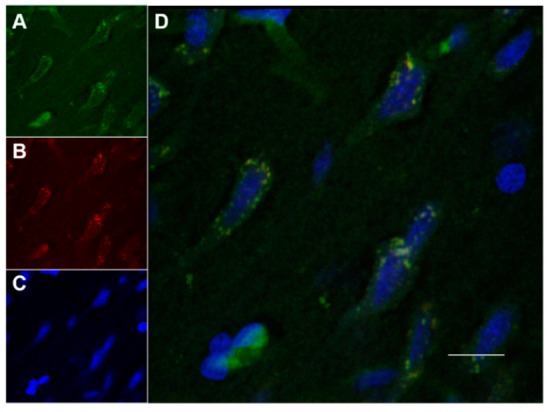
Confocal microscopy images of the parietal cortex of animals submitted to tMCAO after 24 h of reperfusion. (**A**): Cy2 green fluorescence represents NGB. (**B**): Rhodamine red fluorescence detects NPs. (**C**): DAPI blue fluorescence marks cell nuclei. Images are representative microphotographs of samples from 8 experimental animals. (**D**): Merge of images of (**A**–**C**). Scale bar: 10 μm.

Figure 7 and Figure 8 showed confocal microscopic images of histological sections from brains extracted at 2 h and 24 h, respectively, after the onset of reperfusion of rats submitted to tMCAO and injected with NGB–NPs. These images represented individual Cy2 (Figure 7A and Figure 8A), rhodamine (Figure 7B and Figure 8B), and DAPI (Figure 7C and Figure 8C) fluorophores, as well as the merged image (Figure 7D). Cy2 fluorescence revealed the presence of NGB labeled using an antiserum against NGB and identified in green using a secondary antibody linked to Cy2. Rhodamine red vesicles corresponded to NPs, and DAPI labeled the cell nuclei in blue. In the merged images, light-orange/yellow color corresponds to the colocalization of NPs and NGB and, therefore, identifies NPs linked to NGB. These images clearly revealed that NGB linked to NPs had crossed the BBB and had penetrated into the nerve cells as early as 2 h after injection. In addition, our results also indicated that fluorescence remained in the cerebral parenchyma 24 h after NGB–NPs injection. It has been reported in a mouse MCAO model that CCP coupled to recombinant NGB were detected in the brain 4 h after intravenous application [1,21]; on the contrary, despite the fact that tMCAO disrupted the BBB (Yang, et al., 2018), NGB without CPP did not permeate the infarcted brain parenchyma [1,21]. Certainly, the NGB detected in our study should correspond not only to the exogenous (linked to NPs) but also to the endogenous NGB. Nevertheless, the NGB colocalized with NPs, which actually represented the majority of the NGB detected with confocal microscopy, corresponding to exogenous NGB (light-orange/yellow color in the merged images). In this sense, it has been described that NGB is induced in stroke only after 48–72 h of reperfusion either in rodents [1] or humans [61]; therefore, after 2 h and 24 h of reperfusion, endogenous NGB should still be scarce. In relation to the mechanisms that allowed the penetration of the NPs into the cells as well as the release of active NGB into the cytoplasm, futures studies should be specifically designed to elucidate these facts.

**Figure 9:**
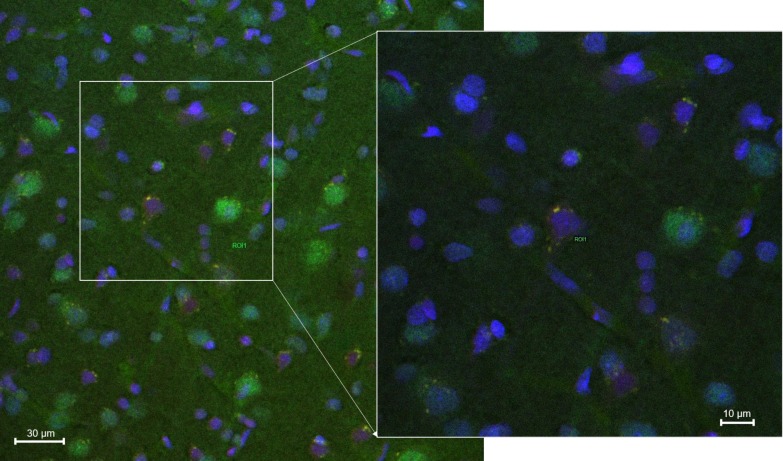
Merged confocal images of the parietal cortex from animals submitted to tMCAO after 2 h of reperfusion. Each image is the result of the overlay of three images taken to visualize NGB (Cy2), NPS (rhodamine), and cell nuclei (DAPI). Images are representative microphotographs of samples from 5 experimental animals. Insert is a zoom taken at higher magnification from the main image. Scale Bar: 30 μm.

**Figure 10:**
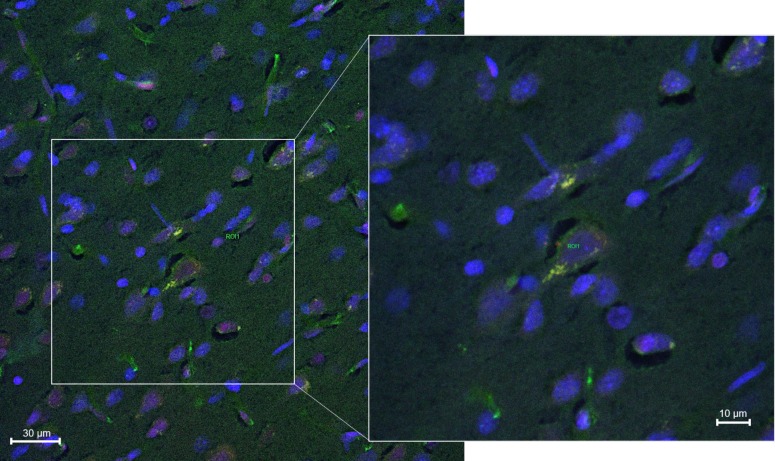
Merged confocal images of the parietal cortex from animals submitted to tMCAO after 24 h of reperfusion. Each image is the result of the overlay of three images taken to visualize NGB (Cy2), NPS (rhodamine) and cell nuclei (DAPI). Images are representative microphotographs of samples from 8 experimental animals. Insert is a zoom taken at higher magnification from the main image. Scale Bar: 30 μm.

The localization of NGB within the cerebral parenchyma in both the 2 h and 24 h reperfusion groups was located in blood capillaries but mainly within the nerve cells (Figure 9 and Figure 10, respectively). These cells that actively capture NGB–NPs once they have crossed the BBB, probably correspond to neurons given their shape and size, in fact, Cai and colleagues [1] have reported that CCA–NGB colocalized with the neuronal marker NSE, but not with the glial marker GFAP. They attributed this feature to the cargo composition, although an alternative explanation could be that NGB might be stable in neurons, while it might be quickly metabolized in glia [21]. Anyway, additional studies will be required to unequivocally confirm the type of nerve cell that stores our NGB–NPs, also elucidating if glial cells that play an important role in the ischemic response (Nedergaard M1, Dirnagl U., 2005) could also be involved.

**Figure 11:**
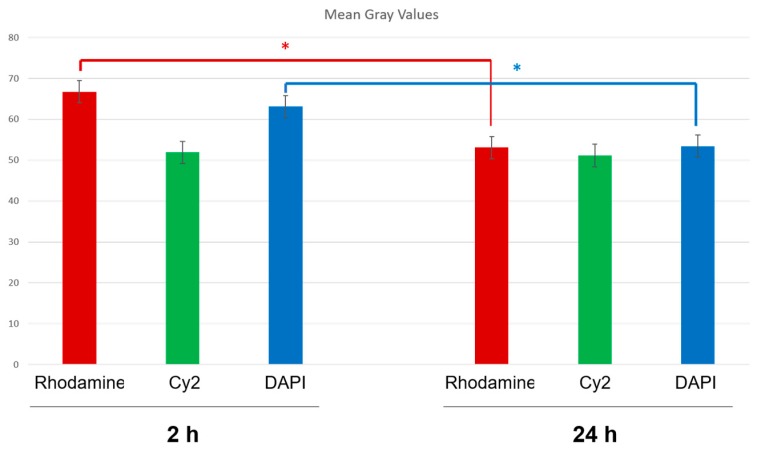
Estimation of the mean grey values of the fluorescent emission from rhodamine (NPs), Cy2 (NGB), and DAPI (nuclei) 2 h and 24 h after MCAO, respectively. Results are the average values of 10 measurements from 8 experimental animals in each group. * *p* < 0.05.

Figure 11 shows the quantification (grey mean values) of Cy2 (NGB), rhodamine (NPs), and DAPI (nuclei) fluorescence in the cerebral parenchyma 2 h and 24 h after the onset of reperfusion, using the Leica advanced software AF (Leica Microsystems, Wetzlar, Germany). This software allows quantify the fluorescence of a determined fluorophore in the microscopic visual field of the histological section selected. The data showed that rhodamine slightly decreased (*p* < 0.05) after 24 h of reperfusion, while NGB remained stable. After 24 h, some rhodamine fluorescence could have been lost; however, as NGB was marked by immunohistochemistry, it was not be severely affected as time elapsed since the NGB–NPs were injected at the onset of the reperfusion. The same result obtained for rhodamine was observed in relation to DAPI, although in this case it could be also related to the changes induced by reperfusion in the infarcted tissue.

## 4. Conclusions

Gelation within an emulsion is an effective method for the encapsulation of the neuroprotective protein NGB. This method, for the first time, has achieved the delivery of NGB linked to NPs from blood to cerebral parenchyma after stroke.

The characterization of these NGB–NPs coated with CH and GT evidenced that they have adequate sizes and charges for use as therapeutic tools. They show good stability for the preparation of an extemporaneous drug formulation since both NPs and NGB are highly stable in lyophilization.

After a stroke, the NGB–NPs injected intravenously at the onset of the reperfusion period, can cross the BBB. They quickly reached the damaged nerve cells, being detected inside cytoplasm after 2 h, and remaining after 24 h of reperfusion.

Overall, the pharmaceutical formulation developed in this study is a delivery system that can successfully carry the neuroprotective oxygen-sensing protein NGB to the damaged ischemic brain after stroke.

## Figures and Tables

**Table 1:** Size, polydispersion, and ζ-potential of the NGB–NPS.

	Size Average (nm)	Polydispersion	ζ-Potential (mV)
**NGB–NPs**	145.77 ± 1.14	0.2628 ± 0.0046	27.55 ± 1.017

**Table 2:** Release kinetics of NGB–NPs.

Order 1	Order 2	Root 2	Root 3
50.41 ± 0.59	49.63 ± 0.55	49.97 ± 0.48	47.51 ± 0.31

**Table 3:** Percentage of lyophilized NGB concentration with respect to a no lyophilized pattern.

	Day 0	Day 15	Day 30
**Percentage of NGB**	95.05%	94.68%	96.07%

**Table 4:** Percentage of lost NGB over time.

Time (Days)	% Lost
**0**	0.00%
**8**	0.36%
**15**	3.70%
**30**	5.45%
**60**	16.32%
**90**	23.97%

**Table 5:** Average size values of lyophilizated NPs.

	Size Average (nm)	Polydispersion	ζ-Potential (mV)
**NGB–NPs**	143.98 ± 2.01	0.2331 ± 0.085	29.11 ± 1.971
